# Peripheral Blood Exosomal miR-184-3p in Methamphetamine Use Disorder: Biomarker Potential and CRTC1-Mediated Neuroadaptation

**DOI:** 10.3390/cimb47070479

**Published:** 2025-06-20

**Authors:** Yan Zhao, Zhuoming Zhao, Qianqian Sun, Hang Su, Tianzhen Chen, Xiaomin Xu, Xiaotong Li, Sai Shi, Jiang Du, Haifeng Jiang, Min Zhao

**Affiliations:** 1 Shanghai Mental Health Center, Shanghai Jiao Tong University School of Medicine, Shanghai 200030, China; wuyiya5@126.com (Y.Z.); zhaozm9916@163.com (Z.Z.); qianqiansun82@126.com (Q.S.); sh3168876@sjtu.edu.cn (H.S.); vomchan@outlook.com (T.C.); xuxiaomin0801@163.com (X.X.); li.xiaotong@zs-hospital.sh.cn (X.L.); peppermiss@sjtu.edu.cn (S.S.); dujiangdou@163.com (J.D.);; 2 Shanghai Key Laboratory of Psychotic Disorders, Shanghai 200030, China; 3 CAS Center for Excellence in Brain Science and Intelligence Technology (CEBSIT), Chinese Academy of Sciences, Shanghai 200030, China

**Keywords:** methamphetamine, exosome, microRNA, biomarker, CRTC1

## Abstract

The neurobiological mechanisms underlying methamphetamine use disorder (MUD) remain elusive, and specific treatment modalities as well as diagnostic markers are scarce. The emergence of exosomes has opened up possibilities for developing diagnostic and assessment biomarkers for neuropsychiatric disorders. Hence, the present study aimed to preliminarily explore the alterations in exosomal miRNA expression in MUD patients and the potential mechanisms involved in MUD. First, miRNA sequencing and RT-qPCR were used to verify the differential expression of peripheral blood exosomal miR-184-3p and miR-4433a-5p in MUD patients. Subsequently, the diagnostic ability of these two miRNAs for MUD was evaluated using ROC analysis. Finally, the regulatory relationship between miRNA-184-3p and its downstream target gene CRTC1 was verified by dual luciferase reporter assay. The results demonstrated that exosomal miR-184-3p and miR-4433a-5p were markedly decreased in MUD patients. However, the expression level of miR-4433a-5p was influenced by anxiety-depressive symptoms. The ROC analysis revealed that the AUCs of exosomal miRNA-184-3p in the training and validation sets of MUD patients were 0.902 and 0.823, respectively. In conclusion, exosomal miR-184-3p levels in peripheral blood may be a potential biomarker for the diagnosis and assessment of MUD, and it may be involved in the pathophysiological process of MUD through the targeted regulation of the CRTC1/CREB pathway.

## 1. Introduction

In 2022, an estimated 30 million people used amphetamine-type substances, accounting for 0.6% of the global population, while 20 million people used ecstasy, representing 0.4% of the global population. The production and trafficking of methamphetamine (MA) have been spreading to new regions globally [1]. Consequently, following opioid abuse, MA abuse has introduced new challenges in the clinical and research fields of addiction. Compared to traditional drugs such as opioids, synthetic drugs represented by MA are characterized by strong addictive potential, significant neurotoxicity, and severe cognitive impairment, posing serious risks to patients’ physical and mental health as well as family and social safety [2,3,4,5]. Opioid use disorder(OUD) can be treated pharmacologically, for example, through methadone replacement therapy which significantly reduces the relapse rate of opioid abuse, but the pathophysiological mechanisms of MA addiction remain unclear, and there are no targeted treatments for MA use disorder (MUD). Therefore, further exploration of MA addiction mechanisms is essential to lay a theoretical foundation for the development of biological diagnostic markers and innovative treatment methods in clinical settings.

Substance use disorder (SUD) is caused by the complex interaction of genetic and environmental factors. miRNAs, involved in post-transcriptional regulation as one of the main forms of epigenetics, widely participate in a series of cellular physiological processes, including cell proliferation and differentiation, signal transduction, cell metabolism, neurotransmission, synaptic vesicle transport and other key biological processes [6,7,8,9], closely related to the occurrence, development, diagnosis and prognosis of mental illnesses [10,11,12,13]. In the central nervous system, miRNAs as key post-transcriptional regulators, control the expression of hundreds of genes related to SUD, thereby directly participating in the regulation of neurotransmission and neural adaptation [14], indicating that the absence or overexpression of miRNAs is not only a result of drug abuse but can also promote the occurrence and development of addiction. In terms of clinical studies, mainly studies from different sample sources from cadaveric brain and peripheral blood, the research strategy is generally to explore the differential expression of miRNAs in addiction-related brain regions and peripheral blood, as well as to explore the differential miRNAs through Receiver Operating Characteristic Curve (ROC) analyses as MUD diagnostic and therapeutic markers. Due to the difficulty in obtaining human specimens, there are fewer cadaveric brain studies of MUD, and the effects of addictive substances on human brain miRNA expression profiles remain largely unknown. One study evaluated miRNA profiles in the ventral tegmental area (VTA) and nucleus ambiguus (NAc) of postmortem brain tissue of 3,4-methylenedioxymethamphetamine abusers, and found that miR-7975 and miR-1202 were significantly down-regulated in these two brain regions [15]. Peripheral blood studies, although more numerous, have yielded inconsistent results. For example, previously we found significantly decreased levels of plasma miR-181a, miR-15b, miR-let-7e and let-7d in patients with MUD [16], but one study had the opposite result and found significantly higher expression levels of let-7b in patients with MUD [17]. Another study exploring the potential of miRNAs in peripheral blood of MUD patients as a non-invasive diagnostic tool for AUD showed a significant increase in miR-9-3p expression in peripheral blood of MUD patients, with an area under the curve (AUC) of the ROC curve ranging from 0.743 [18]. However, there is still a lack of widely recognized MUD-related miRNAs, which may be related to the fact that miRNA expression levels are influenced by various factors, including differences between individuals (such as genetic background, age, gender, lifestyle, etc.), environmental factors (such as infection, stress, inflammation, etc.) and at different stages of the same disease, all of which can affect miRNA expression. Therefore, it is currently challenging to translate miRNA research into clinical applications.

In recent years, the emergence of exosomes has provided potential diagnostic markers and new therapeutic tools for neuropsychiatric diseases. All cells, prokaryotes and eukaryotes release extracellular vesicles (EVs) under normal or abnormal physiological conditions. Based on their origin and biogenesis mechanisms, EVs can be mainly divided into two categories: exosomes and ectosomes [19]. Exosomes are vesicles formed through the endosomal pathway within cells, typically ranging in diameter from 40–160 nm. Depending on the cellular source can contain many cellular components including membrane proteins, cytoplasmic and nuclear proteins, extracellular matrix proteins, metabolites, mRNA, ncRNA and DNA [20,21,22,23]. Studies have found that exosomes are related to immune response, viral pathogenicity, pregnancy, cardiovascular diseases, central nervous system-related diseases and cancer progression [24]. Exosomes are present in all biological fluids and are secreted by all cells, making samples easily obtainable with minimal trauma. Moreover, they can cross the blood-brain barrier (BBB) under specific conditions [25], and have the ability to transfer proteins [26]. The dysregulated expression profiles of exosomal miRNAs in peripheral circulation can reflect pathological and physiological states, giving them the potential to track disease progression through longitudinal sampling. Currently, the focus diseases for exosome diagnostic applications include cardiovascular diseases [27,28], diseases affecting the central nervous system (CNS) [29] and cancer [30]. Exosomes themselves, or as drug carriers, are being actively researched as a therapeutic approach. Compared to liposomes, injected exosomes can efficiently enter other cells [30,31,32], and they are well-tolerated, with exosomes from mesenchymal stem cells (MSCs) and epithelial cells not causing toxicity upon repeated injections in mice [33,34]. MSC-derived exosomes showed no significant side effects and elicited a positive response in a patient with graft-versus-host disease (GVHD) after repeated injections [35].

Currently, a limited number of studies have explored alterations in the expression profiles of peripheral blood exosomal miRNAs in MUD. For instance, one clinical study found that plasma and exosomal miR-320 could serve as potential blood biomarkers for diagnosing MUD, with related target pathways primarily involving cardiovascular diseases, synaptic plasticity and neuroinflammation [36]. However, due to varying disease states (such as during MA use or post-withdrawal) and differing confounding factors (such as accompanying anxiety and depressive symptoms), there is no unified or definitive exosomal miRNA associated with MUD. Therefore, it remains essential to continue exploring changes in exosomal miRNA expression levels and their potential mechanisms in MUD.

Thus, the aim of our study is to investigate the differences in exosomal miRNA expression in MUD patients during the withdrawal period and their potential downstream targets. Additionally, given that depression and anxiety are common symptoms during MUD withdrawal, we also explored the relationship between differentially expressed exosomal miRNAs and anxiety and depressive symptoms. We first isolated exosomes from the peripheral blood of MUD patients and identified exosomal differentially expressed miRNAs (DE-miRNAs) between MUD patients and healthy controls (HC) through miRNA sequencing. Subsequently, by combining the enrichment analysis results of DE-miRNAs and reviewing the literature, we identified miR-184-3p and miR-4433a-5p, which may regulate the CRTC1/CREB pathway, as exosomal DE-miRNAs for RT-qPCR validation. We also established associations between exosomal DE-miRNAs and depression and anxiety scores in MUD patients. Finally, we used receiver operating characteristic (ROC) curve analysis to evaluate the diagnostic capability of DE-miRNAs for MUD.

## 2. Materials and Methods

### 2.1. Study Participants and Ethical Statement

Methamphetamine use disorder (MUD) patients were recruited from the addiction ward of Wuhan Mental Health Center prior to the initiation of pharmacological treatment. Healthy controls (HCs) matched for age and sex were recruited from the community. All participants were enrolled based on the following inclusion criteria: (a) absence of major infectious diseases, other significant chronic illnesses, or relevant family history; (b) provision of informed consent to participate in the study; and (c) diagnosis of MUD in patients according to the Diagnostic and Statistical Manual of Mental Disorders, Fifth Edition (DSM-5) criteria. Exclusion criteria included: (a) current or history of somatic diseases or infectious diseases; and (b) concurrent abuse of another substance. Clinical data, including age, sex, and methamphetamine use history, were collected from all patients using standardized questionnaires. The study protocol was reviewed and approved by the Ethics Committee of Shanghai Mental Health Center (Approval No. 2021-71) and was filed with the Wuhan Mental Health Center.

### 2.2. Scale Administration

Depressive symptoms were evaluated using the Patient Health Questionnaire-9 (PHQ-9), a self-report instrument designed to assess the presence and severity of depressive symptoms. The PHQ-9 consists of nine items, each scored on a 4-point Likert scale ranging from 0 (not at all) to 3 (nearly every day). The total score ranges from 0 to 27, with higher scores indicating more severe depressive symptoms. A score of 0–4 indicates no depressive symptoms, while a score >4 suggests the presence of depressive symptoms [37,38]. Anxiety symptoms were assessed using the Generalized Anxiety Disorder 7-item scale (GAD-7), a validated tool for measuring the presence and severity of anxiety symptoms. The GAD-7 comprises seven items, each rated on a 4-point Likert scale from 0 (not at all) to 3 (nearly every day). The total score ranges from 0 to 21, with higher scores reflecting greater anxiety severity. A score of 0–4 indicates no anxiety symptoms, whereas a score >4 signifies the presence of anxiety symptoms [39,40].

### 2.3. Blood Sample Collection and Preparation

Whole blood samples (5 mL) were collected from all participants using vacuum blood collection tubes containing EDTA as an anticoagulant immediately after enrollment. The anticoagulant-treated blood samples were gently mixed by inverting the tubes several times. Plasma was separated from blood cells within 2 h of collection. Briefly, the blood samples were centrifuged at 800× *g* for 10 min at 4 °C to obtain the supernatant. The supernatant was then transferred to a 2 mL microcentrifuge tube and centrifuged again at 16,000× *g* for 10 min at 4 °C to remove residual cellular debris. The resulting supernatant was carefully transferred to RNase/DNase-free tubes and stored at −80 °C for subsequent analysis.

### 2.4. Isolation of Circulating Exosomes

Exosomes were isolated from samples according to the manufacturer’s protocol using the exoEasy Maxi Kit (Qiagen, Hilden, Germany). Briefly, the sample was mixed with XBP buffer and loaded onto the exoEasy spin column. The mixture was centrifuged at 500× *g* for 1 min, and the flow-through was discarded. The column was then placed back into the collection tube. Next, 10 mL of XWP buffer was added to the column, followed by centrifugation at 3000× *g* for 5 min, and the flow-through was discarded. The column was transferred to a new collection tube, and 400 μL to 1 mL of XE buffer was added. After incubation at room temperature for 1 min, the column was centrifuged at 500× *g* for 5 min to collect the exosomes. The eluate was reloaded onto the column membrane, incubated at room temperature for 1 min, and centrifuged at 3000× *g* for 5 min. The final eluate containing exosomes was transferred to a 1.5 mL microcentrifuge tube and stored at −80 °C for further use.

### 2.5. Transmission Electron Microscopy (TEM) for Exosome Characterization

Exosomes were characterized using transmission electron microscopy (TEM) to confirm their morphology and size distribution. Briefly, a 10-μL aliquot of the exosome sample was applied to a copper grid and allowed to settle for 1 min, after which the excess liquid was blotted with filter paper. Subsequently, 10 μL of phosphotungstic acid was added to the grid and allowed to settle for 1 min, followed by blotting off the excess liquid with filter paper. The grid was then air-dried for several minutes. TEM imaging was performed at 80 kV using a Hitachi H-7650 transmission electron microscope (Hitachi, Tokyo, Japan).

### 2.6. RNA Extraction

Total RNA was extracted from plasma and exosome using miRNeasy Serum/Plasma Kit (217184, Qiagen, Hilden, Germany) according to the manufacturer’s specifications. The yield of RNA was determined using a NanoDrop 2000 spectrophotometer (Thermo Scientific, Waltham, MA, USA), and the integrity was evaluated using agarose gel electrophoresis stained with ethidium bromide.

### 2.7. miRNA Library Preparation and Sequencing

Small RNA libraries were constructed using the TruSeq Small RNA Library Preparation Kit (Illumina, San Diego, CA, USA) following the manufacturer’s protocol. Briefly, 3′ and 5′ adapters were sequentially ligated to the RNA through a series of denaturation and ligation steps, with reactions performed at controlled temperatures using a PCR machine. Reverse transcription and PCR amplification were then carried out to generate cDNA libraries. Sequencing was performed on an Illumina NovaSeq 6000 platform (Illumina, USA) to generate 50 bp single-end reads. All steps were conducted with reagents prepared on ice to ensure stability, and the final libraries were quantified and quality-checked prior to sequencing.

### 2.8. Quantification and Differential Expression Analysis of miRNA

Use Bowtie tools soft, The Clean Reads respectively with Silva database, GtRNAdb database, Rfam database and Repbase database sequence alignment, filter ribosomal RNA (rRNA), transfer RNA (tRNA), small nuclear RNA (snRNA), small nucleolar RNA (snoRNA) and other ncRNA and repeats. The remaining reads were used to detect known miRNA and new miRNA predicted by comparing with known miRNAs from miRbase. Read count for each miRNA was obtained from the mapping results, and Transcripts Per Million (TPM) was calculated.

### 2.9. Prediction and Functional Analysis of miRNA-Targeted Genes

Using miRanda for target gene prediction of differential miRNAs, the score threshold was set to 150 and the energy threshold to −30 kcal/mol. To assess the potential functions of the identified target genes, Gene Ontology (GO) and Kyoto Encyclopedia of Genes and Genomes (KEGG) pathway enrichment analyses were performed. GO enrichment analysis was conducted using topGO 3.12 (https://doi.org/10.18129/B9.bioc.topGO, accessed on 5 April 2020), while KEGG pathway analysis was carried out using KOBAS (http://kobas.cbi.pku.edu.cn, accessed on 5 April 2020). The KEGG database (https://www.kegg.jp/kegg/kegg1.html, accessed on 5 April 2020) was utilized as a reference for pathway annotation.

### 2.10. Real-Time Quantitative RT-PCR

Quantification was performed with a two-step reaction process: reverse transcription (RT) and PCR. Each RT reaction consisted of 0.5 μg RNA, 5 μL of 2× TS miRNA Reaction Mix and 0.5 μL of TransScrip miRNA RT Enzyme Mix, in a total volume of 10 μL. Reactions were performed in a GeneAmp^®^ PCR System 9700 (Applied Biosystems, Foster City, CA, USA) for 60 min at 37 °C, followed by heat inactivation of RT for 5 s at 85 °C. The 10 μL RT reaction mix was then diluted × 10 in nuclease-free water and held at −20 °C.

Real-time PCR was performed using LightCycler^®^ 480 Ⅱ Real-time PCR Instrument (Roche Diagnostics, Mannheim, Germany; manufactured in Rotkreuz, Switzerland) with 10 μL PCR reaction mixture that included 1 μL of cDNA, 5 μL of 2× PerfectStartTM Green qPCR SuperMix, 0.2 μL of universal primer, 0.2 μL of microRNA-specific primer and 3.6 μL of nuclease-free water. Reactions were incubated in a 384-well optical plate (Roche, Basel, Switzerland) at 94 °C for 30 s, followed by 45 cycles of 94 °C for 5 s, 60 °C for 30 s. Each sample was run in triplicate for analysis. At the end of the PCR cycles, melting curve analysis was performed to validate the specific generation of the expected PCR product. The microRNA-specific primer sequences were designed in the laboratory and synthesized by TsingKe Biotech based on the microRNA sequences obtained from the miRBase database (Release 20.0) as follows:hsa-miR-4433a-5p: 5′TCCCACCCCCCACTCCTGT3′, hsa-miR-184-3p: 5′GACGGAGAACTGATAAGGGT3′.The expression levels of microRNAs were normalized to 5S rRNA and were calculated using the 2^−ΔΔCt^ method [41].

### 2.11. The Dual-Luciferase Reporter Assay

The dual-luciferase reporter assay was performed to evaluate the regulatory activity of target sequences. Briefly, the target sequences were cloned upstream of the firefly luciferase (Fluc) gene in the reporter plasmid, while the Renilla luciferase (Rluc) gene in the control plasmid served as an internal control. HEK 293T cells were cultured in DMEM medium supplemented with 10% fetal bovine serum and transfected with the reporter and control plasmids using a lipid-based transfection method. After 48 h, cells were lysed with lysis buffer, and the lysates were centrifuged to obtain supernatants for luciferase activity measurements. Firefly luciferase activity was first measured by adding LAR II buffer, followed by the addition of Stop & Glo buffer to measure Renilla luciferase activity. The relative luciferase activity (RLA) was calculated as the ratio of Fluc to Rluc activity, normalized to the control group.

### 2.12. Statistical Analysis

All data were analyzed using SPSS 24.0 statistical software (IBM, Armonk, NY, USA), and graphs were generated using the GraphPad Prism software 10.0 (GraphPad, San Diego, CA, USA). Quantitative data are expressed as the mean ± standard deviation (SD), while categorical data are represented by proportions or ratios. The Mann-Whitney U test was used to assess non-parametric data. One-way ANOVA was used to analyze the expression levels of exosomal miRNA among the three groups. Spearman’s rank correlation was utilized for correlation analysis. To identify the exosomal miRNA biomarker panel for MUD, receiver operating characteristic (ROC) curve analysis was conducted, and the area under the ROC curve (AUC) was also calculated. For all statistical tests, a *p*-value of less than 0.05 was considered to indicate statistical significance. Significant differences were indicated as * *p* < 0.05, ** *p* < 0.01, and *** *p* < 0.001.

## 3. Results

### 3.1. Characteristics and Clinical Data Analysis of the Study Population

A total of 28 individuals diagnosed with MUD were enrolled in this study, including 25 males (89.3%), with a mean age of 32.07 ± 4.39 years. Additionally, 19 healthy controls (HCs) were recruited, including 14 males (73.7%), with a mean age of 30.00 ± 6.31 years. No significant differences were observed between the two groups in terms of gender and age (*p* > 0.05). However, significant differences were identified in years of education and marital status (*p* < 0.05). The mean PHQ-9 score in the MUD group was 8.50 ± 6.50, and the mean GAD-7 score was 5.96 ± 4.48. The control group exhibited significantly lower mean scores, with a PHQ-9 score of 3.89 ± 3.16 and a GAD-7 score of 1.79 ± 2.15. Statistical analysis revealed significant differences between the two groups in both PHQ-9 and GAD-7 scores (*p* < 0.05). Regarding the pattern of MA use, the mean age of first use in the MUD group was 23.82 ± 5.55 years, the mean MA consumption per occasion was 0.51 ± 0.28 g, the mean duration of MA use was 5.60 ± 3.13 years, and the mean duration of abstinence was 11.47 ± 15.34 months (Table 1).

### 3.2. Transmission Electron Microscopy (TEM) Analysis of Exosomes

**Figure 1:**
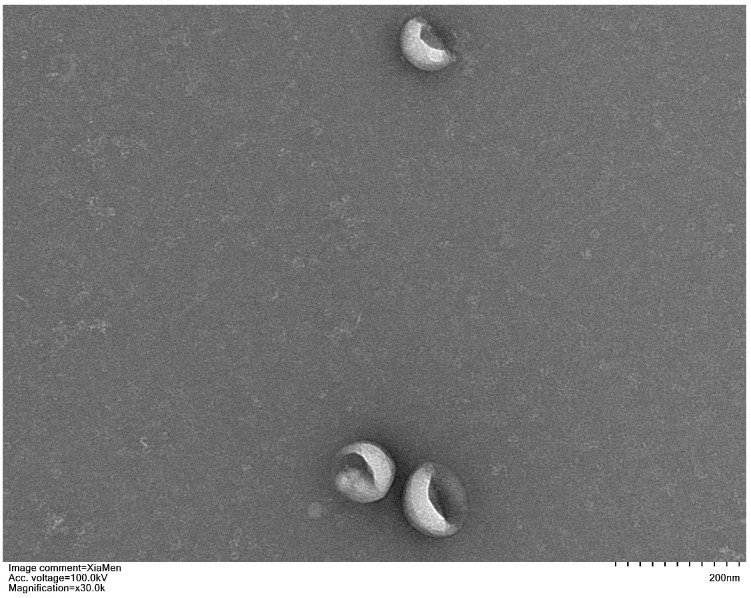
Representative transmission electron micrograph images of exosomes derived from study participants, scale bar = 200 nm.

After isolating exosomes, their morphology was examined using Transmission Electron Microscopy (TEM) to confirm that the obtained nanoparticles were indeed exosomes. The TEM results revealed that exosomes derived from both MUD patients and HC exhibited a characteristic cup-shaped morphology, with a diameter ranging between 50–150 nm (Figure 1).

### 3.3. Differential Expression of Exosomal miRNAs Between MUD and HC

**Figure 2:**
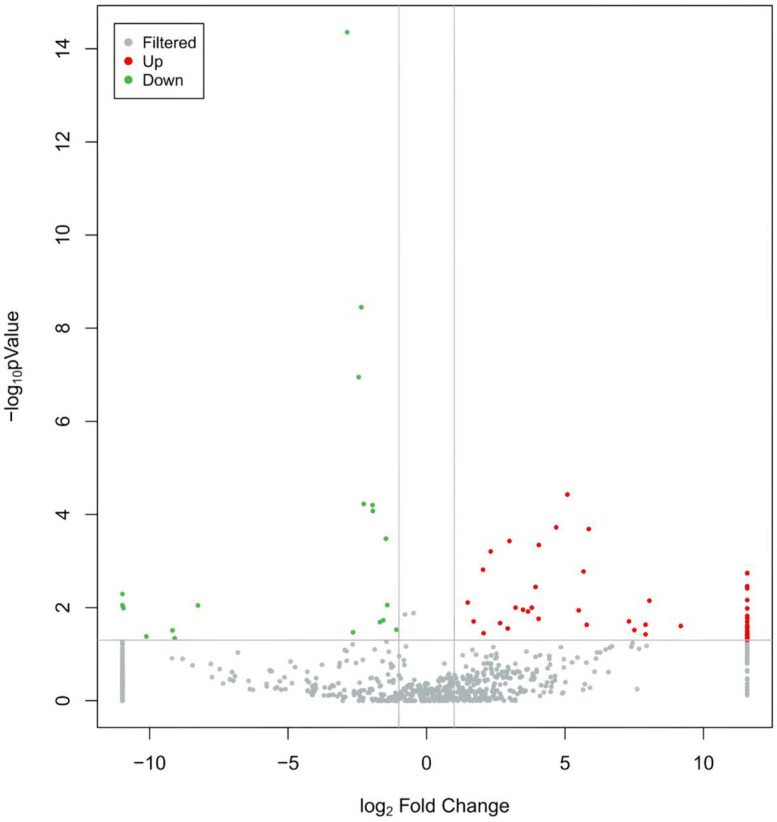
Volcano plot of differentially expressed miRNAs. Notes: The gray represents non-differentially expressed miRNAs, red represents significantly up-regulated differentially expressed miRNAs, and green represents significantly down-regulated differentially expressed miRNAs.

The peripheral blood exosomal miRNAs of 9 MUD patients and 10 age and gender matched HCs were sequenced using RNA-seq. Small RNAs are diverse and include miRNAs, tRNAs (tiRNAs, tRFs), rRNAs, piRNAs, snRNAs, etc. To classify and annotate the small RNAs in the sequencing results, clean reads were sequentially aligned and annotated against the Rfam database, cDNA sequences and species repeat sequence libraries. The length distribution analysis of miRNAs revealed that 20–24 nucleotides were the most common read lengths, with a peak at 22, indicating that mature miRNAs were appropriately enriched during the sequencing library preparation process. To focus on the miRNAs that are ubiquitously abundant in exosome and suitable as biomarker candidates, we selected miRNA candidates with an absolute fold change in expression of ≥2, a nominal FDR (False Discovery Rate) value of ≤0.05 and expression (TPM ≥ 0) across ≥50% of samples. The results showed that compared to the control group, the MUD group had 113 differentially expressed exosomal miRNAs, with 94 upregulated and 19 downregulated (Figure 2, Table S1). Target gene prediction for the differentially expressed miRNAs was performed using miRanda, and 97 out of the 113 differentially expressed miRNAs had downstream target genes, predicting a total of 68,091 target genes and 199,279 binding sites.

### 3.4. Potential Regulatory Roles of DE-miRNAs

**Figure 3:**
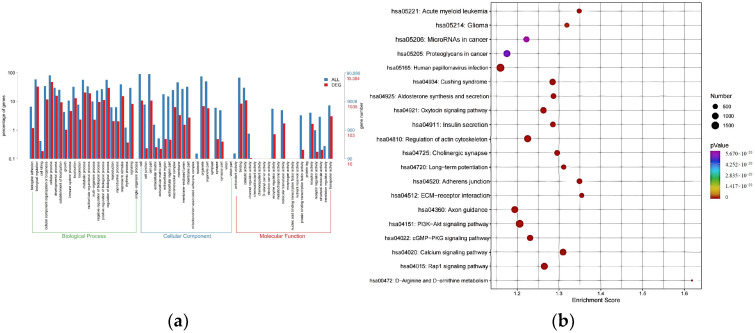
Major categories of GO terms and top 20 significantly enriched KEGG pathways regulated by candidate target genes of differentially expressed miRNAs. (**a**) GO terms in biological process, cellular component and molecular function enriched for DE-miRNAs from MUD vs. HC. (**b**) KEGG pathways enriched for DE-miRNAs from MUD vs. HC. Different color and diameter of the pathway dots represent significance level and gene number respectively.

GO and KEGG pathway enrichment analyses were conducted to elucidate the potential molecular pathways implicated in MUD. GO enrichment analysis revealed that the main enrichments in biological processes included regulation of biological processes, cellular processes, and biological regulation. For cellular components, the main enrichments were associated with cells, cell parts, and organelles. Regarding molecular functions, the main enrichments were observed in catalytic activity, binding, and transporter activity (Figure 3a). KEGG pathway analysis further identified several pathways potentially linked to substance use disorder, such as long-term potentiation, regulation of the actin cytoskeleton, oxytocin signaling pathway and PI3K-Akt signaling pathway. These findings suggest that these pathways may play critical roles in the pathogenesis of MUD (Figure 3b).

### 3.5. Selection of Target Exosomal miRNAs

Based on the results of KEGG pathway analysis, long-term potentiation (LTP) was identified as one of the significantly enriched pathways. Substance abuse alters synaptic function and plasticity in relevant brain circuits to induce long-term behavioral changes, and LTP plays a key role in this process. It has been shown that CRTC1 can maintain late LTP (L-LTP) by promoting CREB-dependent gene transcription (e.g., BDNF), which regulates synaptic plasticity and participates in the substance addiction process [42]. Consistent with this, overexpression of dominant-negative mutants of CRTC1 or knockdown of CRTC1 in hippocampal neurons reduces activity-dependent transcription of CREB target genes, whereas overexpression of wild-type CRTC1 enhances both basal and activity-dependent transcription of CREB target genes. Furthermore, overexpression of dominant-negative CRTC1 mutants impairs the maintenance of L-LTP, whereas overexpression of wild-type CRTC1 enhances L-LTP [43]. Also previous studies have shown that miR-184-3p can directly regulate CRTC1 expression [44]. Therefore, based on a combination of sequencing, target gene prediction, enrichment analysis and previous literature, the differentially expressed exosomes miR-4433a-5p and miR-184-3p with CRTC1 as the target mRNA were selected as candidate miRNAs for further validation in the expanded sample set.

### 3.6. Alteration of Exosomal miR-4433a-5p and miR-184-3p Expression in MUD Patients

**Figure 4:**
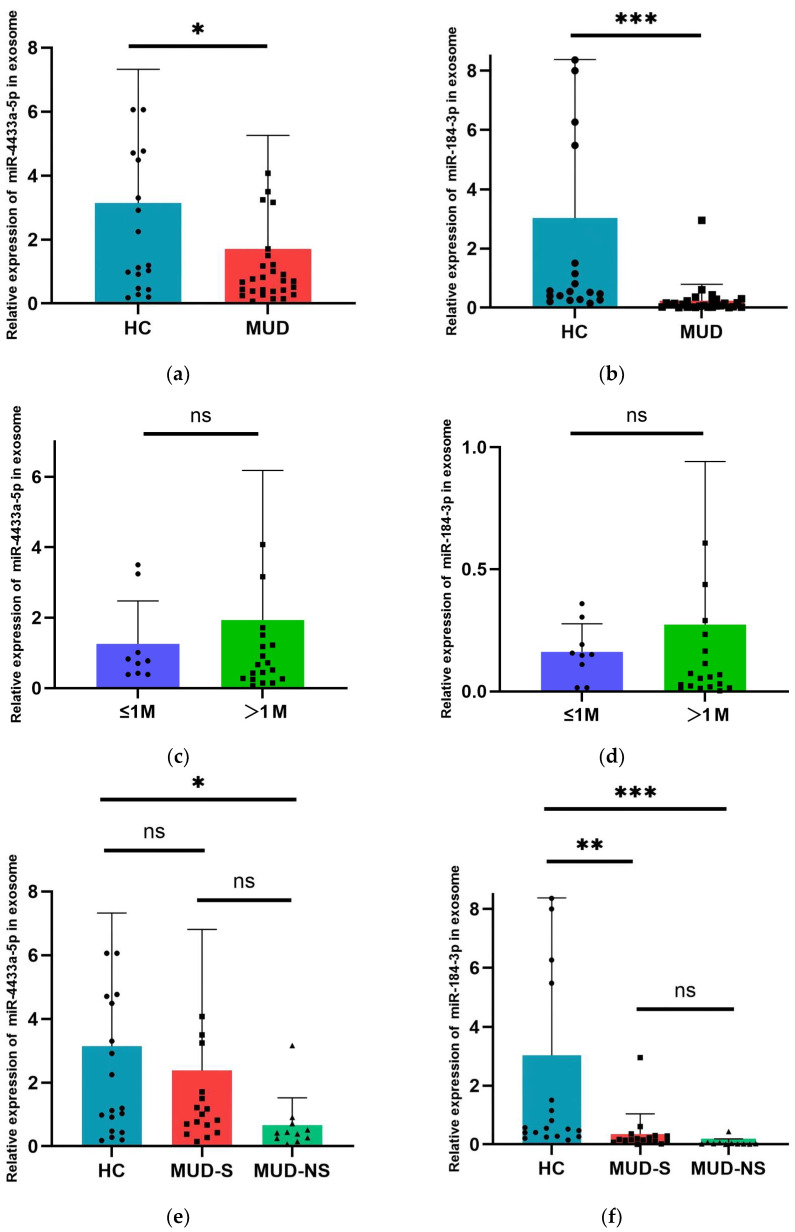
Expression level of 2 selected miRNAs assessed by RT-qPCR. (**a**) Comparison of miR-4433a-5p relative expression levels between MUD and HC. (**b**) Comparison of miR-184-3p relative expression levels between MUD and HC. (**c**) Comparison of miR-4433a-5p relative expression levels between different subgroups in MUD. (**d**) Comparison of miR-184-3p relative expression levels between different subgroups in MUD. (**e**) Comparison of miR-4433a-5p relative expression levels between between the three groups. (**f**) Comparison of miR-184-3p relative expression levels between between the three groups. Data are expressed as the mean ± SD.* *p* < 0.05, ** *p* < 0.01 and *** *p* < 0.001 indicate statistically significant differences compared to baseline. The dots, squares, and small triangles in the figure caption represent the miRNA 2^−ΔΔCt^values of specific individuals in different groups.

We utilized RT-qPCR to validate the expression levels of exosomal miR-4433a-5p and miR-184-3p. The results demonstrated that, compared to the HC group, the expression levels of exosomal miR-4433a-5p and miR-184-3p were significantly downregulated in MUD patients (*p* < 0.05) (Figure 4a,b). Furthermosre, when the MUD patients were stratified into two groups based on the duration of abstinence (≤1 month and >1 month), no significant differences were observed in the expression levels of exosomal miR-4433a-5p and miR-184-3p between these two subgroups (Figure 4c,d).

Significant differences in PHQ-9 and GAD-7 scores were found between MUD patients and healthy controls. Consequently, MUD patients were categorized into two groups: MUD with anxiety and depressive symptoms (MUD-S, PHQ-9 or GAD-7 score >4) and MUD without anxiety and depressive symptoms (MUD-NS, PHQ-9 or GAD-7 score ≤4). The expression levels of miR-4433a-5p and miR-184-3p were compared between these subgroups in MUD and the HC group. The results indicated that there was a significant difference in the expression level of miR-4433a-5p between the HC group and the MUD-NS group, with no significant difference observed between the healthy control group and the MUD-S group, or between the MUD-NS and MUD-S groups(Figure 4e). For miR-184-3p, significant differences in expression levels were found between the HC group and both the MUD groups with and without anxiety and depressive symptoms, while no significant difference was observed between the MUD-NS and MUD-S groups(Figure 4f).

### 3.7. Correlation Between Exosomal DE-miRNAs and the Characteristics of MA Use

**Figure 5:**
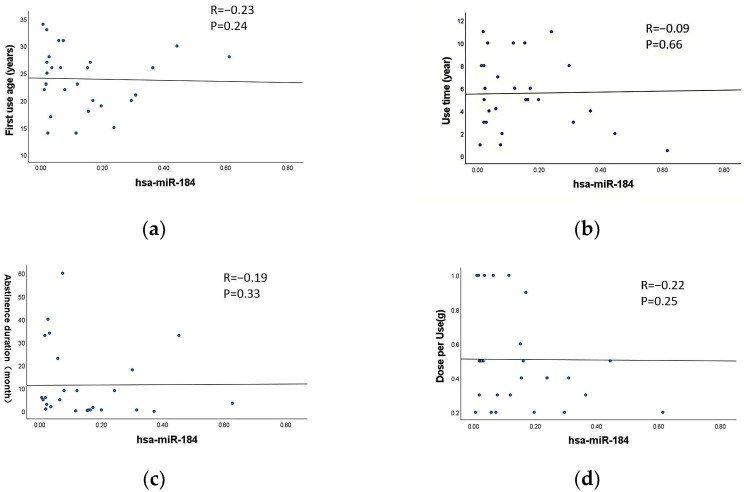

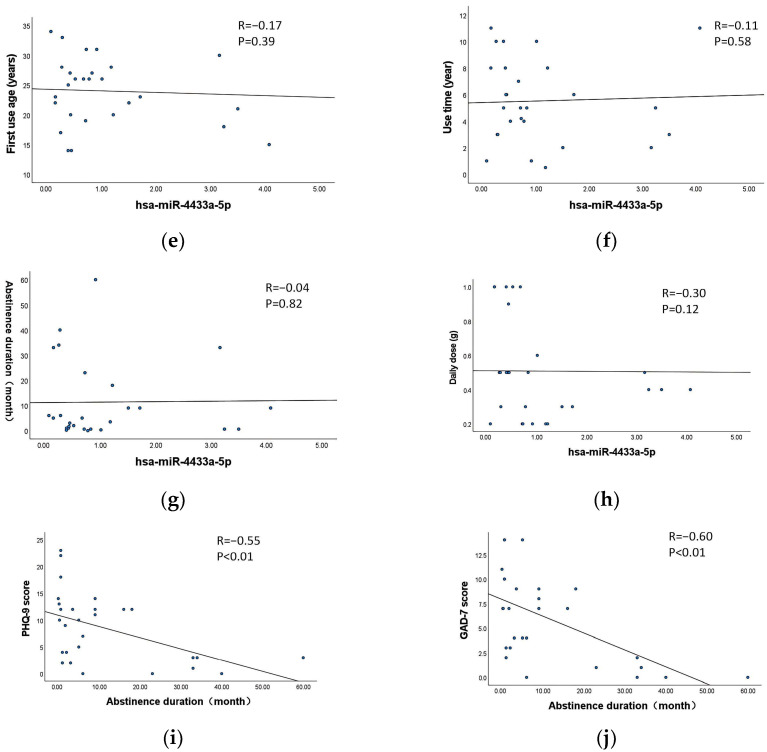
Correlation between exosomal DE-miRNAs and the characteristics of MA use. (**a**–**d**) Correlation between miR-184-3p and first use age, use time, abstinence duration and dose per use, respectively. (**e**–**h**) Correlation between miR-4433a-5p and first use age, use time, abstinence duration and dose per use, respectively. (**i**) Correlation between withdrawal duration and the scores of PHQ-9. (**j**) Correlation between withdrawal duration and the scores of GAD-7.The dots in the figure represent individual sample data points, and the line represents the fitted linear regression line.

Correlation analysis was performed to assess the relationship between the characteristics of MA use (first use age, use time, abstinence duration and dose per use) and the expression levels of exosomal miR-4433a-5p and miR-184-3p in MUD patients. The results indicated no significant correlation between the expression levels of exosomal miR-4433a-5p and miR-184-3p and any characteristics of MA abuse parameters (*p* > 0.05) (Figure 5a–h). This suggests that the expression levels of these miRNAs are may not influenced by the specific characteristics of MA use.

### 3.8. Correlation Between Withdrawal Duration and the Scores of PHQ-9 and GAD-7

Correlation analysis was conducted to examine the relationship between the abstinence duration and PHQ-9 as well as GAD-7 scores in MUD patients. The results revealed a significant negative correlation between the abstinence duration and both PHQ-9 and GAD-7 scores (*p* < 0.01) (Figure 5i,j). Specifically, longer abstinence durations were associated with lower PHQ-9 and GAD-7 scores.

### 3.9. Diagnostic Accuracy of miR-4433a-5p and miR-184-3p as Diagnostic Biomarkers

**Figure 6:**
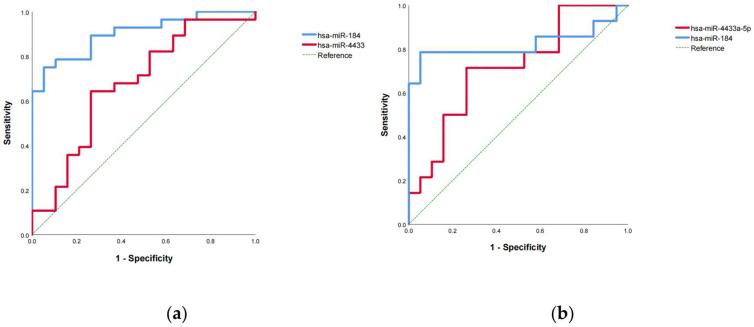
ROC analysis demonstrated that miR-184-3p exhibited a strong diagnostic performance for the identification of patients with MUD. (**a**) ROC curve analysis of exosomal miR-4433a-5p and miR-184-3p in the training set. (**b**) ROC curve analysis of exosomal miR-4433a-5p and miR-184-3p in the validation set.

To evaluate the diagnostic potential of exosomal miR-4433a-5p and miR-184-3p for MUD, ROC curves were constructed using the expression levels (ΔΔCt values) of these miRNAs, and the area under the curve (AUC) was calculated. ROC curve analysis demonstrated that peripheral blood exosomal miR-4433a-5p and miR-184-3p exhibited good discriminative ability between MUD patients and healthy controls, with AUC values of 0.677 (*p* < 0.05) and 0.902 (*p* < 0.01), respectively (Figure 6a). The optimal cutoff values, determined by the Youden index, were 0.13 for miR-4433a-5p and 2.32 for miR-184-3p.

Subsequently, the expression levels of exosomal miR-4433a-5p and miR-184-3p from a validation set of MUD patients (Table S2) and the training set of healthy controls were combined to re-plot the ROC curves and calculate the AUC. The results confirmed that exosomal miR-4433a-5p and miR-184-3p maintained good discriminative ability, with AUC values of 0.714 (*p* < 0.05) and 0.823 (*p* < 0.01), respectively (Figure 6b). Using the established cutoff values, diagnostic performance metrics were calculated. For miR-4433a-5p (cutoff = 0.13), the sensitivity was 71.43%, specificity was 47.37%, accuracy was 57.58%, positive predictive value (PPV) was 50%, negative predictive value (NPV) was 69.23%, false positive rate was 52.63%, and false negative rate was 28.57%. The F1 score of the model was 58.82%. For miR-184-3p (cutoff = 2.32), the sensitivity was 64.29%, specificity was 100%, accuracy was 84.85%, PPV was 100%, NPV was 79.17%, false positive rate was 0%, and false negative rate was 35.71%. The F1 score of the model was 78.08%.These findings suggest that exosomal miR-184-3p has strong diagnostic potential for MUD with high specificity and accuracy, while miR-4433a-5p has average diagnostic performance.

### 3.10. Dual-Luciferase Reporter Assay Validation

**Figure 7:**
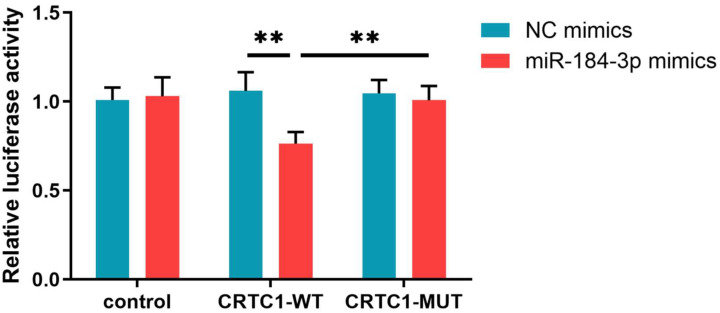
Dual-Luciferase Reporter Assay Results of miRNA-184-3p and CRTC1.NC mimics: Negative Control mimics; CRTC1-WT; CRTC1 Wild Type; CRTC1-MUT; CRTC1 Mutant Type; control: empty vector.** * p* < 0.01 indicates statistically significant differences compared to baseline.

The previous results suggest that the altered expression level of exosomal miRNA-4433a-5p in MUD is influenced by anxiety-depressive symptoms and has a low potential as a diagnostic marker for MUD, thus only validating the regulatory relationship between miRNA-184-3p and CRTC1. The results demonstrated that, compared to the NC mimics + CRTC1-WT group, the CRTC1 expression level was significantly decreased in the miR-184-3p mimics + CRTC1-WT group. In contrast, no significant differences were observed between the NC mimics and miR-184-3p mimics groups in either the control or CRTC1-MUT groups. Furthermore, CRTC1 expression was significantly lower in the miR-184-3p mimics + CRTC1-WT group compared to the miR-184-3p mimics + CRTC1-MUT group. These findings indicate that miR-184-3p can directly target and downregulate the expression of CRTC1. This provides strong evidence that miR-184-3p functions as a regulatory molecule by binding to the 3′ untranslated region (3′ UTR) of CRTC1, thereby suppressing its expression (Figure 7).

## 4. Discussion

In this study, we initially conducted sequencing of peripheral blood exosomal miRNAs in both MUD patients and healthy controls. Subsequent functional analysis and literature review were performed to identify differentially expressed miRNAs for validation. Through RT-qPCR, we confirmed significant differential expression of exosomal miR-4433a-5p and miR-184-3p in abstinent MUD patients. Correlation and ROC analyses suggested that miR-184-3p has potential as a diagnostic biomarker for MUD. Furthermore, its role in MUD may be mediated through the targeted regulation of CRTC1/CREB pathway.

In this study, the recruited MUD patients and healthy controls were matched in terms of age and gender, but significant differences were observed in years of education and occupational status (*p* < 0.05), which is consistent with previous research findings [45].The PHQ-9 and GAD-7 scores in the MUD group indicated mild anxiety and depressive symptoms in the MUD patients, whereas the healthy controls had no anxiety and depressive symptoms, with a significant difference between the two groups (*p* < 0.05).Anxiety is a common symptom during MA abuse and withdrawal [2,46,47], with the prevalence of comorbid anxiety disorders in MUD reaching as high as 30.2% [48]. Moreover, comorbid anxiety increases treatment difficulty and the risk of relapse [49]. A significant correlation also exists between MA abuse and depressive symptoms, as the likelihood of developing depression is several times higher in individuals with MUD compared to those without. Additionally, MA abuse, particularly MUD, is a potential risk factor for depression, which aligns with the high rates of depressive disorders observed among MUD patients seeking treatment [50]. However, some reviews have noted that a clear causal relationship between MA abuse and depressive symptoms, as well as between MA abuse and anxiety symptoms, has not been established. Further longitudinal studies are required to elucidate these causal relationships [51], during which various mediating factors, moderators and covariates related to anxiety/depressive symptoms and MA abuse, such as biological and environmental factors, need to be considered [52]. However, the results of our study suggest that the longer the duration of abstinence, the less severe the MUD-related anxiety and depression symptoms may be, consistent with previous studies [46,53]. Regarding treatment, existing reviews have explored interventions for MUD comorbid with anxiety disorders or symptoms, including pharmacological treatments (e.g., antipsychotics, antidepressants, non-amphetamine stimulants like modafinil and dietary supplements), psychosocial interventions (e.g., motivational enhancement) and exercise therapy. However, the efficacy of these treatments varies, and there is a lack of unified therapeutic recommendations [54]. A significant proportion of MA abusers seek medical services due to anxiety and depressive symptoms. The longitudinal study will provide data on how medical services targeting emotional symptoms can reduce MA abuse and thus maximise the effectiveness of medical interventions [55].

This study validated the significant downregulation of exosomal miR-4433a-5p and miR-184-3p in abstinent MUD patients. Additionally, the expression levels of exosomal miR-4433a-5p and miR-184-3p were compared among three groups: healthy controls, MUD patients without anxiety and depressive symptoms and MUD patients with anxiety and depressive symptoms. The results revealed that, compared to the control group, miR-4433a-5p expression was significantly downregulated in MUD patients without anxiety and depressive symptoms, while a downward trend was observed in MUD patients with anxiety and depressive symptoms, though it was not statistically significant. This suggests a potential interaction between miR-4433a-5p expression and the manifestation of anxiety and depressive symptoms. In contrast, miR-184-3p expression was significantly downregulated in both MUD patients with and without anxiety and depressive symptoms compared to the control group, with no significant difference between the two MUD subgroups. This indicates that miR-184-3p expression levels may have a weaker association with the manifestation of anxiety and depressive symptoms.

Currently, there is a lack of research on the exosomes miR-4433a-5p and miR-184-3p in SUD. Existing studies on miR-184-3p have confirmed its regulatory relationship with CRTC1, demonstrating that miR-184-3p inhibits CRTC1 expression [44,56], which aligns with the findings of this study. CRTCs (CREB Regulated Transcription Coactivators), discovered by Vadim Iourgenko et al., are coactivators of the cAMP response element-binding protein (CREB). They enhance CREB activity and promote CREB-dependent gene transcription [57,58]. The CRTC family consists of three members (CRTC1, CRTC2, and CRTC3), which are evolutionarily conserved, with functional homologs identified in Drosophila melanogaster and Caenorhabditis elegans [59,60]. CRTC1 is predominantly expressed in the brains of humans and rodents [43] and plays a crucial role in neuronal dendritic growth and development, synaptic plasticity, LTP, memory consolidation and reconsolidation [57,58]. When cells are stimulated (e.g., by cAMP or Ca^2^⁺ signaling), protein kinase A (PKA) or calcineurin is activated, triggering CRTC1 dephosphorylation and nuclear translocation. CRTC1 then binds to CREB at relevant promoters, enhancing CREB activity and promoting CREB-dependent gene transcription. Knockout of CRTC1 reduces the expression of CREB-regulated downstream genes and impairs neuronal dendritic growth and synaptic plasticity [42,43,61], whereas overexpression of CRTC1 enhances the expression of CREB-regulated downstream genes [57]. The CREB-dependent gene BDNF is involved in maintaining L-LTP in the mouse hippocampus [42]. In cultured hippocampal neurons, overexpression of a dominant-negative mutant of CRTC1 or knockdown of CRTC1 expression reduces activity-dependent transcription of CREB target genes, while overexpression of wild-type CRTC1 promotes both basal and activity-induced transcription of CREB target genes. Overexpression of the dominant-negative CRTC1 mutant inhibits the maintenance of L-LTP, whereas overexpression of wild-type CRTC1 enhances L-LTP [43]. Therefore, it is proposed that CRTC1 maintains L-LTP by inducing CREB-dependent gene transcription, regulates synaptic plasticity and thereby modulates learning and memory, participating in the process of substance addiction. This study found that exosomal miR-184-3p is significantly downregulated in MUD patients, and according to the regulatory relationship, CRTC1 expression is upregulated, which is consistent with previous studies on changes in CRTC1 expression in SUD. Repeated excessive cocaine use is considered a significant risk factor for the loss of control over self-administration behavior. Under unrestricted access to cocaine, the self-administration behavior in rats is regulated by miR-212 in the striatum, a process mediated by enhancing the CRTC1/CREB pathway [62].

Current research on miR-4433a-5p primarily focuses on its potential as a biomarker in the field of oncology, including testicular germ cell tumors [63], colon cancer [64], gallbladder cancer [65] and thyroid cancer [66]. However, miR-4433a-5p was found to target and downregulate the PIK3R2 gene, which in turn regulates the PI3K/Akt signaling pathway [67]. KEGG pathway analysis indicates that the target genes of DE-miRNAs are enriched in the PI3K/Akt signaling pathway, regulation of the actin cytoskeleton and LTP. A large number of DE-miRNAs were found in rat striatum after MA exposure, and subsequent Western Blot verified that the expression levels of PI3K, Akt and p-Akt in the PI3K/Akt signaling pathway were significantly elevated in the striatum, suggesting that exposure to MA affects the activity of the PI3K/Akt signaling pathway [68]. The activated PI3K/Akt signaling pathway can modulate dynamic changes in the actin cytoskeleton [69], which serves as one of the structural foundations of dendritic spines. The morphology of dendritic spines influences synaptic efficacy, the generation and maintenance of LTP and other processes. Numerous studies have found that the regulation of the actin cytoskeleton is associated with SUD, including cocaine and amphetamines [70,71]. For example, following chronic amphetamine exposure in mice, changes in miR-29a/b were observed in specific brain regions. These changes downregulated Arpc3 (actin-related protein 2/3 complex subunit 3, Arp2/3 complex), reducing mushroom-shaped dendritic spines and increasing filopodia-like protrusions in hippocampal neurons, suggesting that miR-29a/b influences synaptic structure and function through actin cytoskeleton remodeling [71]. Therefore, it is proposed that miR-4433a-5p may participate in MUD by regulating the PI3K/Akt signaling pathway-actin cytoskeleton dynamics- synaptic plasticity.

In investigating the correlation between DE-miRNAs and MA abuse characteristics, no significant associations were found. Furthermore, no statistically significant differences in the expression levels of these miRNAs were detected between patients with abstinence durations of less than one month and those with abstinence durations exceeding one month. These findings suggest that alterations in exosomal miR-4433a-5p and miR-184-3p expression levels may persist in the peripheral blood of MUD patients over prolonged periods. Subsequent ROC analysis was employed to evaluate the diagnostic potential of exosomal miR-4433a-5p and miR-184-3p across distinct MUD patient cohorts. The results indicate that exosomal miR-184-3p has good diagnostic properties, highlighting its potential use as a biomarker for MUD. Exosomal miRNAs are known for their remarkable stability in extracellular environments, maintaining consistent expression levels in blood, urine and other biofluids over extended periods. Emerging evidence suggests that exosomal miRNAs may traverse the blood-brain barrier, positioning them as promising molecular markers for investigating psychiatric disorders and SUD [72,73,74]. Preliminary studies have begun to explore the feasibility of exosomal miRNAs as biomarkers in SUD. For instance, ROC analysis of differentially expressed exosomal hsa-miR-451a and hsa-miR-21a in the plasma of MUD patients yielded area under the curve (AUC) values of 0.966 and 0.861, respectively, indicating their potential for predicting MUD [75]. Additionally, research has investigated the therapeutic potential of exosomes in neuroprotection, neuronal regeneration and modulation of neuroplasticity, with a focus on their utility as drug delivery vehicles [31,76]. For example, a recent study demonstrated that exosomes derived from mesenchymal stem cells (MSCs), which contain miRNAs, mRNAs and proteins, ameliorated MA-induced cognitive impairment by enhancing hippocampal neurogenesis and suppressing neuroinflammatory responses [77]. These findings collectively underscore the potential of exosomal miRNAs as both novel therapeutic agents and peripheral biomarkers for MUD. However, the complexity of the mechanism of action of exosomal miRNAs increases the complexity of their use as diagnostic markers, and their study in substance usd disorder remains to be deepened.

Our study has several limitations that should be acknowledged. First, although we validated the altered expression levels of miR-184-3p in MUD patients and proposed its potential involvement in MUD through the regulation of the CRTC1/CREB pathway, behavioral validation is still lacking. The specific mechanisms underlying this regulatory relationship remain to be confirmed through further experimental studies. Second, the relatively small sample size and the use of the same healthy control dataset for both training and validation in the ROC analysis may limit the diagnostic efficacy of miR-184-3p. While the model demonstrated high sensitivity and specificity in the validation set, this design could lead to overly optimistic results and a risk of overfitting. Future studies should validate the model using an independent healthy control dataset to ensure its generalizability and practical applicability.Third, the cross-sectional design of this study makes it difficult to establish a causal relationship between MUD and the observed changes in DE-miRNAs. Longitudinal clinical studies or animal experiments would be valuable for elucidating the mechanisms underlying these associations and providing deeper insights into the role of miR-184-3p and related pathways in MA addiction. Addressing these limitations in future research will enhance the robustness and translational potential of the findings.

## 5. Conclusions

This study revealed alterations in the expression profiles of peripheral blood exosomal miRNAs in MUD patients during the abstinence period. These DE-miRNAs may participate in the pathophysiological processes of MUD through various biological mechanisms. Furthermore, our findings highlight the potential of exosomal miR-184-3p as a biomarker for MUD and suggest its possible role in MUD through the regulation of the CRTC1/CREB pathway. These results provide new insights into the molecular mechanisms underlying MUD and offer a potential target for future diagnostic and therapeutic strategies.

## Figures and Tables

**Table 1:** Demographic and Clinical Characteristics of MUD Patients and Healthy Controls.

Variable	MUD (N = 28)	HC (N = 19)	t/χ^2^	*p*-Value
Age (years)	32.07 ± 4.39	30.00 ± 6.31	−1.89	0.059
Gender (Male)	25 (89.29%)	14 (73.68%)	1.95	0.163
Marital Status			3.81	0.149
Single	13 (46.43%)	11 (57.89%)		
Married	10 (35.71%)	8 (42.11%)		
Divorced	5 (17.86%)	0 (0%)		
Years of Education	11.68 ± 2.34	14.53 ± 2.50	−3.43	0.001
Employment Status (Employed)	20 (71.43%)	19 (100.0%)	6.54	0.011
First Use age (years)	23.82 ± 5.55	-	-	-
Use time (years)	5.60 ± 3.13	-	-	-
Abstinence Duration (months)	11.47 ± 15.34	-	-	-
Dose per Use (g)	0.51 ± 0.28	-	-	-
PHQ-9 Score	8.50 ± 6.50	3.89 ± 3.16	−2.52	0.012
GAD-7 Score	5.96 ± 4.48	1.79 ± 2.15	−3.25	0.001

Notes: Data are presented as mean ± standard deviation or frequency (percentage). MUD: Methamphetamine Use Disorder; HC: Healthy Controls; PHQ-9: Patient Health Questionnaire-9; GAD-7: Generalized Anxiety Disorder-7.

## Data Availability

The datasets used and/or analyzed during the current study are available from the corresponding author on reasonable request.

## Supplementary Materials

The following supporting information can be downloaded at: https://www.mdpi.com/article/10.3390/cimb47070479/s1.

## Abbreviations

The following abbreviations are used in this manuscript:

MUD	Methamphetamine use disorder
HC	Healthy control
RT-qPCR	Reverse transcription quantitative polymerase chain reaction
ROC	Receiver operating characteristic curve
AUC	Area under the curve
MA	Methamphetamine
SUD	Substance use disorder
PHQ-9	Patient Health Questionnaire-9
GAD-7	Generalized Anxiety Disorder 7-item scale
LTP	Long-term potentiation
CREB	cAMP response element binding protein
CRTC1	CREB regulated transcription coactivator 1
