# Supplementary material for: Peripheral Blood Exosomal miR-184-3p in Methamphetamine Use Disorder: Biomarker Potential and CRTC1-Mediated Neuroadaptation
Source: Curr Issues Mol Biol. 2025 Jun 20;47(7):479. doi: 10.3390/cimb47070479 (PMC12293370; doi:10.3390/cimb47070479)
Supplement: Supplementary file 1 [file cimb-47-00479-s001.zip › cimb-3655324-supplementary.pdf]

Table S1. Differential gene expression analysis: log2(FoldChange) and P-values between HC and MUD groups.

miRNA_id	HC(n=10)	MUD(n=9)	log2(FoldChange)	pValue
hsa-let-7a-5p	167929.848273639	23008.0265595798	-2.86764953	0.000
hsa-miR-10b-5p	86283.2458431779	16870.928099612	-2.354541108	0.000
hsa-let-7f-5p	59191.9648951815	10824.3425944755	-2.451121941	0.000
hsa-miR-184	116.072046395418	3940.98676508971	5.08546442929212	0.000
hsa-let-7c-5p	24179.8628592915	5023.72302767715	-2.26697723	0.000
hsa-let-7b-5p	441958.499563028	114295.885901922	-1.951137431	0.000
hsa-miR-126-3p	35766.0159803507	9317.56801965602	-1.940564073	0.000
hsa-miR-199b-5p	45.5132056983096	1167.65703005494	4.68118756541804	0.000
hsa-miR-215-5p	24.6663999616181	1429.33403393782	5.85665213101487	0.000
hsa-miR-10a-5p	57597.1487289231	20849.342881716	-1.46599548	0.000
hsa-miR-199a-3p	497.278456598879	3964.79848471937	2.99512170492587	0.000
hsa-miR-192-5p	404.442127944928	6709.50718823365	4.05220161893116	0.000
hsa-miR-143-3p	3032.1381578503	15100.9406672723	2.31623102486566	0.001
hsa-miR-21-5p	4593.01597743034	18859.6541880346	2.03778951560527	0.002
hsa-miR-4433a-5p	47.7824307018877	2434.23384865186	5.6708437134915	0.002
novel679_mature	0	0.311113738190661	Inf	0.002
novel260_mature	0	0.330106067839172	Inf	0.002
novel14_mature	0	90.5504711148659	Inf	0.003
hsa-miR-200b-3p	78.1337399054733	1196.01563066807	3.93614676287374	0.004
novel515_mature	0	100.683010020906	Inf	0.004
hsa-miR-30c-2-3p	143.13598355892	0	Inf	0.005
novel189_mature>novel1223_mature	0	86.9464841111251	Inf	0.007
hsa-miR-188-5p	0.198734580872282	52.6046341388197	8.04820315626028	0.007
hsa-miR-203a-3p	1690.25503295434	4737.59038719722	1.48691252536364	0.008
hsa-miR-125a-5p	12529.7599764498	4679.20358234401	-1.421023874	0.009
hsa-miR-125a-3p	147.803177787052	0.0730303521355373	-10.98289348	0.009
hsa-miR-424-3p	147.627652622391	0.483077605198712	-8.255492294	0.009
hsa-miR-375-3p	469.542576919633	4355.11961942744	3.21338445617877	0.010
hsa-miR-9-5p	63.5868909764559	885.985401651657	3.80048165134486	0.010
hsa-miR-3605-3p	144.134659278895	0.0730303521355373	-10.94663349	0.010
hsa-miR-6777-5p	0	0.315769291807509	Inf	0.010
novel1517_mature	0	0.238083386055123	Inf	0.010
hsa-miR-152-3p	95.0534347777933	1062.67948218435	3.4828239569164	0.011
hsa-miR-381-3p	7.22367844111431	325.425394934117	5.4934493541873	0.011
hsa-miR-145-5p	58.2785905027371	738.224042251602	3.66302083296731	0.012
novel270_mature	0	121.6430013	Inf	0.015
novel843_mature	0	168.129122699454	Inf	0.017
hsa-miR-145-3p	31.1174605283016	513.697223419841	4.04512204278523	0.017

hsa-let-7e-5p	11641.655986836	3943.16386178803	-1.561870722	0.019
hsa-miR-362-5p	0.778625043999703	123.473037244956	7.30905157131969	0.020
hsa-miR-148a-3p	29309.5923872091	95310.3450919108	1.70125990997847	0.020
novel1426_mature	0	20.8215181636736	Inf	0.020
hsa-miR-320a-3p	6593.25479413662	2054.15879906704	-1.68244312	0.020
hsa-miR-199a-5p	141.017299122923	889.585925593755	2.65726180861832	0.021
hsa-miR-147b-3p	0.600307301903123	144.214293447858	7.90829722907707	0.023
hsa-miR-3168	4.21240915375772	231.824608804011	5.78224433721602	0.023
hsa-miR-5193	0	89.3268961619429	Inf	0.024
hsa-miR-4466	0.0369714515510618	21.4256946715163	9.17871457846283	0.025
novel520_mature	0	0.356637425873295	Inf	0.025
novel1555_mature	0	0.355470538262871	Inf	0.025
hsa-miR-6891-5p	0	50.6026924221449	Inf	0.026
novel524_mature>novel593_mature>novel840_mature>novel975_mature	0	61.8442488483799	Inf	0.026
novel348_mature	0	14.3118782524323	Inf	0.026
novel539_mature>novel624_mature	0	1.44077438367264	Inf	0.027
novel1092_mature>novel1118_mature	0	24.2488140416795	Inf	0.027
hsa-miR-124-3p	0	20.3154763621964	Inf	0.027
hsa-miR-432-5p	312.294071527246	2383.94496972302	2.93237384628793	0.028
hsa-miR-99b-5p	11744.1919098015	5524.79915722554	-1.087953524	0.030
hsa-miR-660-5p	0.645363021806599	117.225199679536	7.50495610494312	0.030
hsa-miR-505-5p	107.636277694251	0.186379138788947	-9.1737083	0.031
novel1159_mature	0	47.0648260237732	Inf	0.031
hsa-miR-183-5p	1834.33590372186	291.16712519289	-2.65533847	0.034
novel175_mature>novel195_mature	0	17.1092616169747	Inf	0.034
novel298_mature	0	10.8682450828902	Inf	0.034
novel233_mature>novel1203_mature	0	13.3671733948833	Inf	0.034
novel514_mature	0	10.4680491339453	Inf	0.034
novel127_mature	0	12.0106374156061	Inf	0.034
novel786_mature	0	10.4444914341545	Inf	0.035
hsa-miR-205-5p	221.908854445173	925.897347667111	2.06088501967404	0.036
novel124_mature	0	15.0644117571796	Inf	0.036
novel1437_mature	0	15.0644117571796	Inf	0.036
novel1469_mature	0	15.774556061276	Inf	0.036
novel886_mature	0	15.774556061276	Inf	0.036
novel228_mature	0	37.430821343124	Inf	0.036
novel123_mature>n	0	15.628495357005	Inf	0.037

ovel144_mature>novel149_mature>novel167_mature>novel782_mature				
novel354_mature	0	13.5307743623333	Inf	0.037
novel1233_mature	0	27.5841125407641	Inf	0.037
novel7_mature	0.0692133641661598	16.6443359099433	7.90976496470527	0.038
novel1408_mature	0	23.5568018395428	Inf	0.039
novel580_mature>novel746_mature	0	24.2292880425631	Inf	0.040
novel1455_mature	0	24.1360984731686	Inf	0.040
novel955_mature	0	24.1360984731686	Inf	0.040
hsa-miR-509-3p	0	21.7660408037889	Inf	0.040
novel1512_mature	0	23.8565297649852	Inf	0.040
novel504_mature	0	21.8063592383067	Inf	0.041
novel248_mature>novel282_mature	0	21.5267905301233	Inf	0.042
hsa-miR-511-5p	81.1611649352598	0.0730303521355373	-10.11807767	0.042
novel667_mature	0	21.060842683151	Inf	0.042
hsa-miR-5088-5p	0	20.874463544362	Inf	0.042
hsa-miR-499b-5p	0	20.2221365586007	Inf	0.043
novel1021_mature	0	18.7109442310302	Inf	0.043
novel1205_mature	0	19.5698095728394	Inf	0.043
novel178_mature	0	18.3583451707112	Inf	0.045
novel1498_mature	0	9.64000648189092	Inf	0.045
novel1378_mature	0	16.8471528431407	Inf	0.045
novel379_mature	0	17.7060181849499	Inf	0.045
novel1004_mature	0	9.31725505646741	Inf	0.045
novel1485_mature	0	17.519639046161	Inf	0.045
novel355_mature	0	9.13320969289931	Inf	0.046
hsa-miR-204-5p	90.812425830711	0.166219921530011	-9.093652604	0.046
novel555_mature>novel568_mature>novel581_mature>novel588_mature	0	15.915257149196	Inf	0.046
novel381_mature	0	8.58107360219502	Inf	0.046
novel13_mature	0	88.1093579688511	Inf	0.046
novel1320_mature	0	16.5877433522162	Inf	0.047
novel165_mature	0	16.3081746440328	Inf	0.047
novel467_mature	0	8.39849049558678	Inf	0.047
novel549_mature	0	15.749037227666	Inf	0.048
novel964_mature	0	15.4694685194826	Inf	0.048
novel927_mature	0	14.6307623949323	Inf	0.049
novel1282_mature	0	14.2580041173544	Inf	0.050

novel571_mature	0	14.2580041173544	Inf	0.050
novel583_mature	0	14.2580041173544	Inf	0.050
novel434_mature	0	14.1648145479599	Inf	0.050

log2(FoldChange): Logarithm base 2 transformed fold-change of expression in MUD relative to HC.

Table S2. Demographic and Clinical Characteristics of the validation set of MUD patients

Variable	MUD (N=14)
Age (years)	44.07 ± 3.54
Gender (Male)	10 (71.4%)
Marital Status	
Single	2 (14.29%)
Married	6(42.86%)
Divorced	6(42.86%)
Years of Education	11.64 ± 2.71
Employment Status (Employed)	4 (28.57%)
First Use age (years)	34.36 ± 6.10
Use time (years)	5.84 ± 4.35
Abstinence Duration (months)	35.11± 30.96
Dose per Use (g)	0.76 ± 0.80
miR-4433a-5p ($2^{-\Delta\Delta Ct}$)	1.23 ± 1.54
miR-184-3p ($2^{-\Delta\Delta Ct}$)	1.55 ± 3.91
